# How to define and assess the clinically significant causes of hematuria in childhood

**DOI:** 10.1007/s00467-022-05746-4

**Published:** 2022-10-19

**Authors:** Orsolya Horváth, Attila J. Szabó, George S. Reusz

**Affiliations:** 1st Department of Pediatrics, Semmelweis University, 53-54 Bókay János Street, Budapest, 1083 Hungary

**Keywords:** Hematuria, Urine, Glomerular Diseases, Red blood cell, Differential diagnosis

## Abstract

Given the wide diversity of causes of hematuria, ranging from simple urinary tract infections with rapid recovery to severe glomerulonephritis with fast decline in kidney function, it is essential to recognize the underlying disease. The first objective of the assessment is to determine whether the cause of the hematuria is medically significant. The combination of hematuria with proteinuria, the presence of hypertension, or worsening kidney function can represent signs of progressive kidney disease. Differentiating the various causes of hematuria is often simple and obvious based on the clinical signs and gross appearance of the urine. However, in some instances, additional non-invasive investigations, such as ultrasound imaging, urinary red cell morphology, measurement of calcium and other solutes in the urine, evaluation of kidney function, and protein excretion, are needed to elucidate the nature of the hematuria. Taking a detailed family history can help in establishing the underlying cause in cases of familial hematuria. On the other hand, the decision to perform a kidney biopsy in children with asymptomatic hematuria remains a challenging issue for clinicians. Ultimately, the frequency of diagnosis of glomerular involvement causing hematuria may depend on the threshold for performing a kidney biopsy. The following review will focus on the diagnostics of hematuria, starting with difficulties regarding its definition, followed by various means to differentiate between urinary, glomerular, and other causes, and finally reviewing the most common diseases that, due to their frequency or their effect on kidney function, present a diagnostic challenge in everyday practice.

## Introduction

The appearance of bloody urine is a concern for parents, children, and even the medical professional. Bloody urine noticeable to the naked eye is recognized quickly; in contrast, hematuria can go unrecognized if it does not stain the urine, that is, it remains microscopic.

Since the causes of hematuria are very diverse, ranging from simple urinary tract infections with rapid recovery to mechanical trauma and severe glomerulonephritis with rapid decline in kidney function, it is essential to recognize the underlying disease and treat it accordingly.

The following review will focus on the diagnostics of hematuria starting with the difficulties regarding its definition, followed by various means to differentiate between urinary, glomerular, and other causes, and finally reviewing the most common diseases that, because of their frequency or their effect on kidney function, present a diagnostic and therapeutic challenge in everyday practice.

## Definitions and detection of hematuria

Hematuria is defined as the presence of erythrocytes (ERYs) in the urine. While definitions can vary, it is usually characterized by > 5 ERYs per high-power field (HPF) upon microscopic examination. In another recommendation, the threshold is 3 or more ERYs per HPF for microscopic hematuria [1]. Urinalysis using a flow cytometer is a new technique useful for confirming hematuria as well as other urinary morphological components. This aspect of the urinalysis is discussed in greater detail in the diagnostic procedures section (see below).

Urine blood test strips can be used as an alternative screening test for hematuria given they are as sensitive as urine sediment examination to detect blood [2]. However, more false-positive tests are generated since the reaction between hemoglobin and the chromogen tetramethylbenzidine used for testing is highly sensitive but not specific. Dipstick testing can detect 1 to 2 ERYs per high-power field. Common causes for a false-positive blood test include the presence of the structurally similar myoglobin, the presence of vitamin C, or contamination with iodine-containing antiseptics. False-negative test strips are rare; thus, a negative test reliably rules out hematuria [3].

The color of urine and the morphology of red blood cells may help in differentiating between possible underlying diseases. Reddish urine alone does not definitively signify hematuria.

### False hematuria or colors mimicking gross hematuria

Pigments and other compounds in certain foods (including beets, berries, and food colorings) and drugs (sulfonamides, ibuprofen, salicylates, phenothiazines, metronidazole, phenolphthalein, chloroquine, deferoxamine, etc.) can change the color of urine [4].

### Macroscopic or gross hematuria

The term gross hematuria refers to visibly bloody urine. It should be noted that 1 ml of blood per liter is sufficient to discolor the urine. Bright red urine, visible clots, or crystals with intact normal ERYs are signs of urinary tract bleeding. Cola-colored urine, ERY casts, and distorted ERYs indicate glomerular disease (see below). Absence of ERYs in urine suggests hemoglobinuria or myoglobinuria [5]. However, ERYs may hemolyze in hypoosmotic urine stored for a longer time, in which case erythrocyte ghosts can be detected under high-magnification light microscopy. This also draws attention to the importance of proper microscopic examination in the recognition of hematuria.

### Microscopic hematuria (MH)

The more common microscopic hematuria means that ERYs are detectable only by direct testing with a urine dipstick, or by direct microscopic visualization following centrifugation.

### Isolated microscopic hematuria (IMH)

Isolated microscopic hematuria is defined as “microscopic hematuria present in mid-stream urine *on more than one occasion*, and unrelated to exercise, trauma or menstruation in the absence of proteinuria, hypertension or kidney impairment at presentation” [6]. Although previously considered a benign condition, it is now recognized that IMH may be associated with an increased risk of kidney failure in the long term [6, 7]. A family history can clarify hereditary causes of hematuria [8].

The main categories causing hematuria in childhood are shown in Table 1 [9–11]. The most significant causes in clinical practice are highlighted in bold, and subsequently discussed in greater detail in the ensuing sections.

**Table 1:** Main categories causing hematuria in childhood (*ANCA*, antineutrophil cytoplasmic antibodies; *DGKE*, diacylglycerol kinase-epsilon; *EHEC*, enterohemorrhagic *Escherichia coli*; *GN*, glomerulonephritis; *IgAVN*, IgA vasculitis nephritis) [9–11]

1. Causes of glomerular hematuria a) Glomerular diseases due to immune-mediated damage to the glomerular structure Most common IgA nephropathy (IgAN) IgAVN (former: Henoch-Schönlein purpura associated glomerulonephritis) Post-infectious nephritis Other nephritises Primary glomerulonephritises (e.g., membranoproliferative GN, membranous GN, C3GN, etc.) Secondary nephritises due to systemic diseases (like systemic lupus erythematosus, ANCA vasculitis, etc.) b) Glomerular diseases due to an inherited abnormality of basement membrane collagens Alport syndrome: X-linked, autosomal, and digenic c) Glomerular diseases due to thrombotic microangiopathy Hemolytic uremic syndrome (HUS) EHEC induced, *Streptococcus pneumoniae*-related HUS, H1N1 and influenza-related HUS Atypical HUS (complement gene mutations, complement factor H antibody, DGKE mutations, cobalamin C defects) HUS with coexisting disease condition (malignancy, solid organ and stem cell transplantation, drug-induced) Thrombotic-thrombocytopenic purpura (TTP)	
2. Causes of postglomerular hematuria a) Hematuria associated with crystal formation Hypercalciuria and other crystallurias Nephrolithiasis (NL) b) Hematuria associated with mechanical damage Trauma Severe hydronephrosis c) Hematuria associated with cyst formation Autosomal dominant polycystic kidney disease (ADPKD) Solitary kidney cyst d) Hematuria associated with vascular damage Nutcracker syndrome (NCS) Hemangioma Arteriovenous malformation Renal vein thrombosis e) Tubulointerstitial nephritis (TIN) (infectious, immune mediated) f) Medications (cyclophosphamide, aspirin, anticoagulants) g) Tumor	
3. Other extra-renal systemic causes of hematuria a) Coagulopathies b) Hemoglobinopathies	

Distinguishing the various causes of hematuria is often simple and obvious based on clinical signs and gross appearance of the urine. However, additional noninvasive investigations may be required to clarify the nature of the hematuria, such as assessment of urinary red blood cell morphology, determination of urinary protein and solute excretion, and assessment of kidney function and kidney morphology by ultrasound imaging. Specific tests are required to distinguish between the different etiologies causing glomerular damage (activity of the complement system, autoantibodies such as dsDNA, antineutrophil cytoplasmic antibodies (ANCA)). In selected cases, an endoscopic examination or a kidney biopsy can provide additional information for the diagnosis.

It should also be borne in mind that different disorders may occasionally present with similar symptoms. Gross hematuria associated with upper respiratory tract infections is a sign of IgA nephropathy (IgAN), but it can also occur in other nephritises and in certain stages and forms of Alport syndrome (AS). In addition, in some cases, multiple etiologies may be detected, such as the mesangial presence of IgA deposits in steroid-sensitive nephrotic syndrome or acute poststreptococcal glomerulonephritis.

In the following, we briefly review some elements of our diagnostic arsenal, such as the role of erythrocyte morphology, the importance of determining the extent of proteinuria, the role of hypercalciuria, the importance of ultrasound imaging as well as the more invasive procedures such as cystoscopy and kidney biopsy in the diagnosis.

## Diagnostic procedures in the investigation of hematuria

The first objective is to establish whether the hematuria is due to a medically significant cause, and in conjunction with the latter, an important consideration for general pediatricians is when to consult the pediatric nephrologist [8]. Furthermore, it is especially important for children to avoid painful and unnecessary examinations.

### Differentiation between glomerular and postglomerular hematuria

**Fig. 1:**
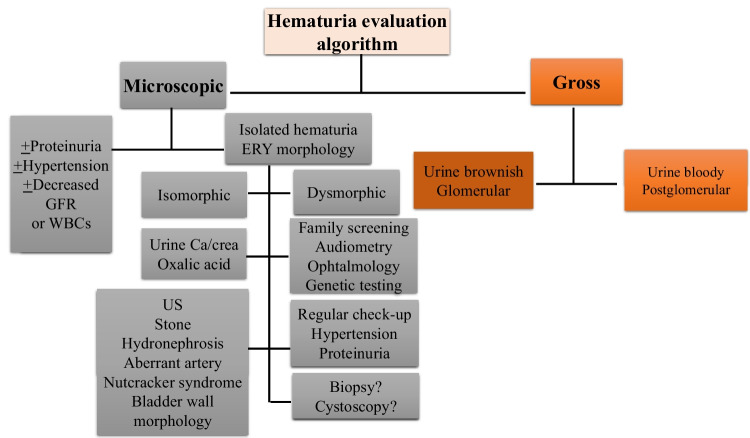
Diagnostic approach in the classification of hematuria in childhood (for details please refer to the text). GFR, glomerular filtration rate; WBCs, white blood cells; crea, creatinine; ERY, erythrocyte; US, ultrasound

The first step in distinguishing the various causes of hematuria is to determine whether the blood is of glomerular or postglomerular origin. An approach summarizing the diagnostic steps is proposed in Fig. 1.

#### Evaluation of urinary erythrocyte morphology

**Fig. 2:**
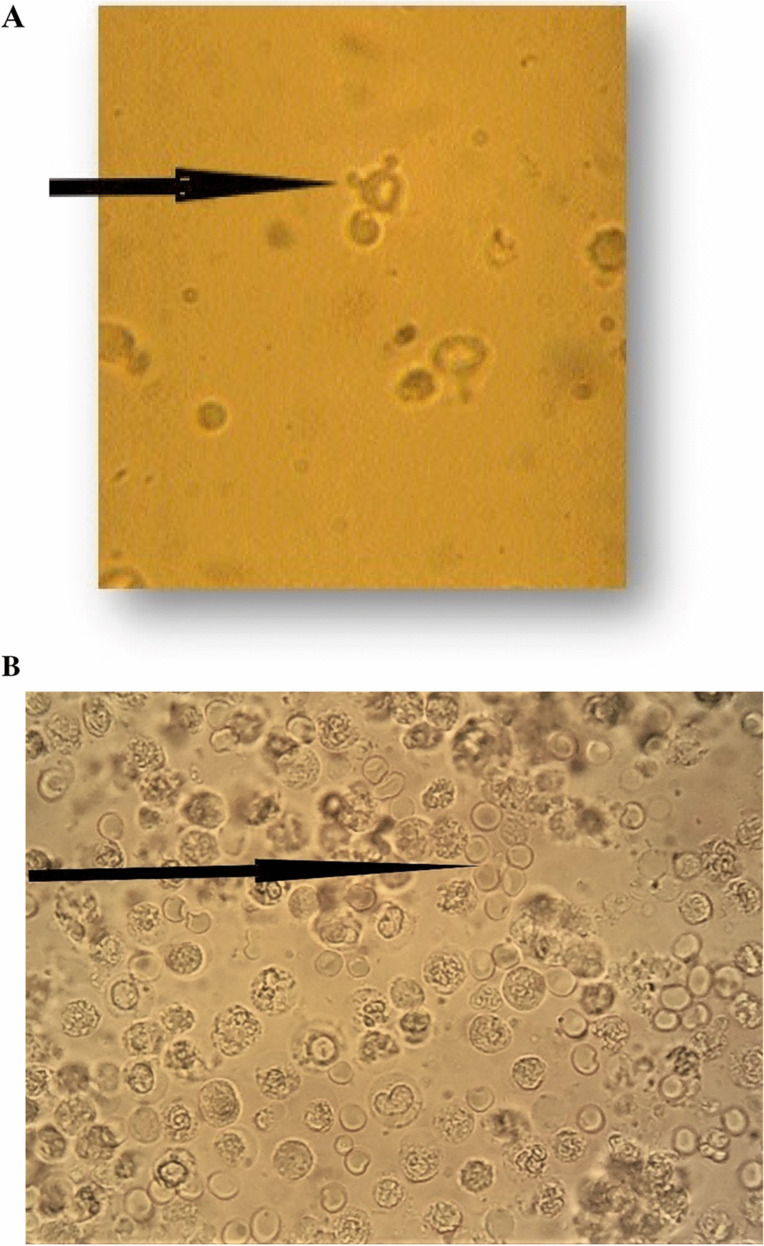
Red blood cell morphology in the urine. **A** Acanthocytes. Irregular erythrocytes with disrupted basement membrane and vesicles on outer surface. (arrow: acanthocyte, also designated as Mickey Mouse cell). **B** Urinary tract infection with leucocytes and isomorphic red blood cells (arrow)

The morphological classification of urinary erythrocytes was introduced into the diagnostic routine in the 1980s to distinguish glomerular from urological hematuria. Evaluation of urinary erythrocyte morphology (UEM) is most useful in identifying patients with glomerular IMH [12]. The most easy-to-understand criterion for dysmorphic cells is “doughnut-like cells with one or more blebs” with additional morphological signs such as budding and partial membrane loss, changes in the shape of red cells, and the average size of blood cells (Fig. 2). In a detailed methodological review, a total of four microscopic criteria were proposed to define IMH as being glomerular: ≥ 40 dysmorphic erythrocytes alone, ≥ 5% acanthocytes alone, erythrocytic casts, and ≥ 40 dysmorphic erythrocytes associated with ≥ 5% acanthocytes [12]. However, a generally accepted system of criteria for the precise evaluation of ERY morphology is still awaited.

The mechanism leading to dysmorphic ERY formation was extensively studied in the 1980–1990s [13]. Dysmorphic ERY can occur as they move through the glomerular basement membrane, traveling through gaps in the kidney capillary wall to reach the Bowman capsule and the tubuli, containing concentrated acidic urine [13–15]. This may be the origin of glomerular hematuria in patients with glomerulonephritis, as well as in common noninflammatory forms of glomerular disease, including Alport syndrome [16, 17].

Overall, examination of red blood cell morphology is an important technique, the optimal assessment requires phase-contrast microscopy; however, the phase-contrast microscope is not available everywhere, and with an adequate magnification and expertise it can also be judged using a simple light microscope [18]. It is not an exclusive means of determining the origin of hematuria, as results may change over time or vary with the degree of hematuria, etc., such that the test should be repeated and supplemented with other non-invasive procedures. All these and the subjective nature of the technique are important limitations of the method.

Urine testing with flow cytometers is a new technical development suitable for confirming hematuria. These instruments are calibrated such that their results are comparable to those of a high-magnification microscopic examination. In addition, the device-specific normal values are also displayed with the results. Flow cytometers have also been used to differentiate the glomerular and non-glomerular origin of hematuria, albeit with conflicting results [19]. While the sensitivity of the method has been found to be low for differentiation in previous studies, newer devices appear to be more accurate in this regard [20]. Nonetheless, extensive clinical trials are still lacking and the final assessment should be made by an experienced and knowledgeable medical eye [19, 20].

#### Combination of proteinuria and hematuria

The combination of hematuria and proteinuria can be a sign of progressive kidney disease; hence, a more detailed investigation is needed. This includes evaluation of the causes listed in Table 1 when supported by clinical signs, such as evaluation for antibodies to autoimmunity, assessment of the complement system, specific infections such as hepatitis C virus, and revealing familiarity if present [21].

Urinary protein excretion in excess of 4 mg/m^2^ per hour is considered abnormal in children [22]. Nephrotic range proteinuria (heavy proteinuria) is defined as ≥ 40 mg/m^2^ per hour and is always indicative of kidney disease. Since a 24-h urine collection may be challenging in children, urinary protein/creatinine ratio on a spot urine sample (uP/Cr) may be used as an alternative, with the following thresholds for nephrotic range proteinuria according to KDIGO 2021: uP/Cr ≥ 2000 mg protein/g creatinine (> 200 mg/mmol) or 3 + on urine dipstick [22, 23]. It is preferably performed on a first morning specimen. However, the difficulty is not in detecting nephrotic proteinuria, but in determining the cutoff value at the lower end of the proteinuria spectrum, and to properly define the latter to separate minor glomerular abnormalities from other significant glomerular changes. The normal value for this ratio is < 0.2 mg protein/mg creatinine (< 20 mg protein/mmol creatinine) in children older than 2 years of age and < 0.5 mg protein/mg creatinine (< 50 mg protein/mmol creatinine) in infants and toddlers from 6 to 24 months [22].

In addition, there are a number of conditions associated with transient proteinuria that may interfere with the diagnosis. For example, urinary tract infections (UTIs) are often associated with positive dipstick urinalysis for proteinuria, and occasionally hematuria may accompany leukocyturia (Fig. 2B) [24]. Positive strip tests can occur due to the reaction of the protein test strip with leukocytes and bacterial proteins; test strip hematuria may be due to red blood cells entering the urinary tract through the capillaries of the inflamed mucosa [24]. Proteinuria is a well-characterized feature of febrile UTI and other febrile diseases of non-kidney origin [25]. Therefore, the presence of proteinuria should be confirmed by repeated measurements in light of clinical symptoms. In the presence of elevated protein excretion or other symptoms of kidney disease (hypertension, kidney impairment), a more detailed nephrological work-up is needed [25].

### Role of ultrasound

**Fig. 3:**
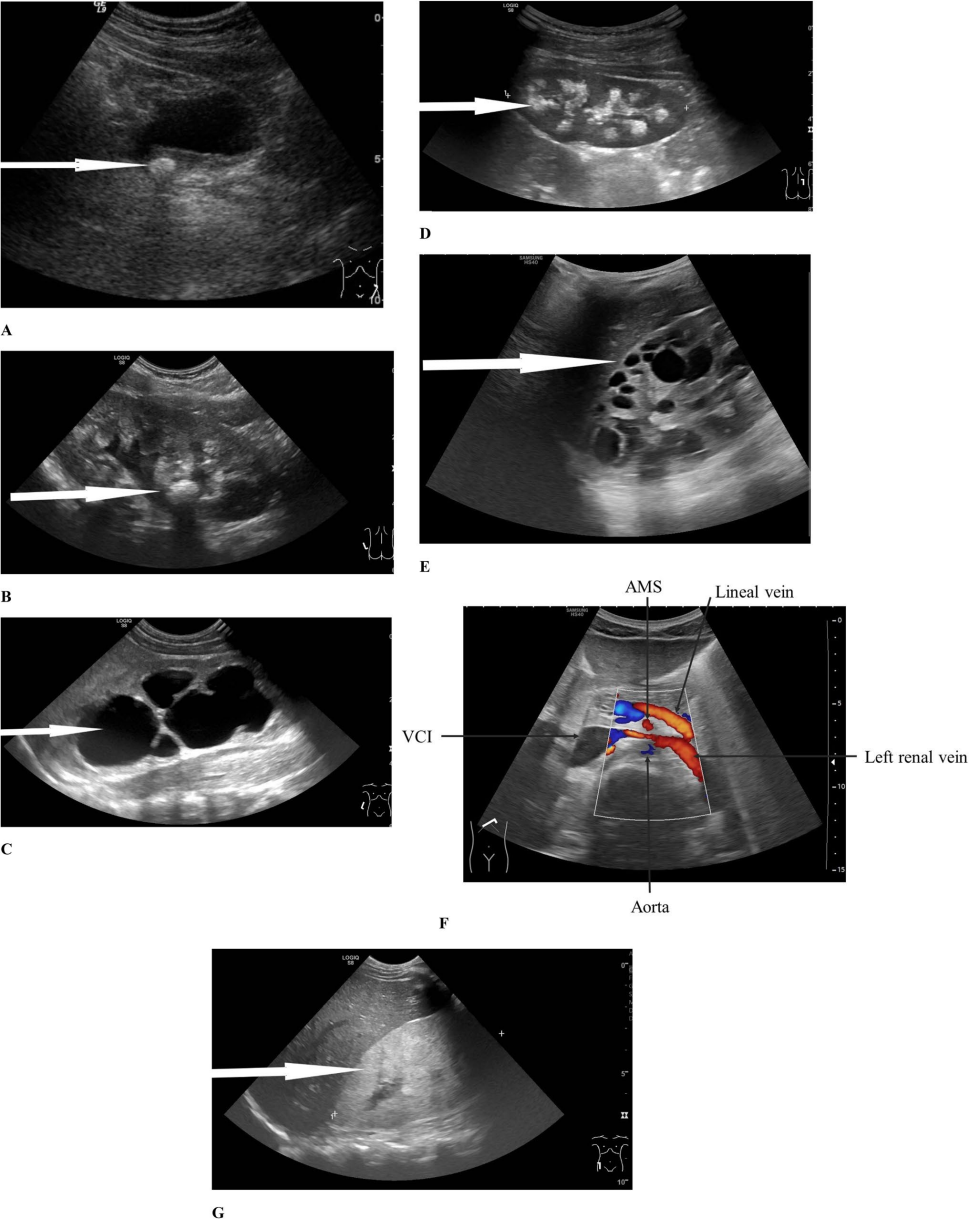
Typical ultrasound images in the setting of hematuria in childhood. **A** Juxtavesical ureteral stone with acoustic shadowing (arrow). **B** Nephrolithiasis (arrow: kidney stone). **C** Hydronephrosis due to pyeloureteral stenosis (arrow: the enlarged pyelon). **D** Nephrocalcinosis (arrow: deposition of calcium salts in papillae of the kidney). **E** Autosomal dominant polycystic kidney disease (ADPKD) (arrow: typical cysts in ADPKD). **F** Nutcracker syndrome (extrinsic compression of the left renal vein by the superior mesenteric artery and aorta; arrows: AMS, superior mesenteric artery; VCI, inferior vena cava). **G** Increase in kidney size and inhomogeneous parenchymal hyperechogenicity including areas of the cortex and medulla in nephrotic syndrome (arrow: hyperechogenic kidney)

Ultrasound is the first and foremost important imaging technique in pediatric nephrology. It should be performed by an experienced pediatric radiologist who will systematically assess the size of the kidney, the morphology of the urinary tract, the echogenicity of the parenchyma, and the perfusion of the kidneys to avoid overlooking any detail. All measurements should be compared with normalized pediatric standard values. Ultrasound examination can detect stones, signs of urinary tract infection, tumors, vascular malformations, hydronephrosis, and kidney cysts in the context of hematuria. Increased parenchymal echogenicity in addition to an increase or decrease in corticomedullary differentiation may be observed in diffuse kidney parenchymal diseases [26]. In the case of autosomal dominant polycystic kidney disease (ADPKD), sonography has a key role in monitoring disease progression [27]. In children with the suspicion of kidney stones, ultrasound should be the first diagnostic imaging modality performed, while low-dose computed tomography (CT), the standard modality used in adults, is rarely required in pediatrics [28]. Kidney Doppler ultrasound is a basic tool to detect nutcracker syndrome [29]. Examples of typical ultrasound images in pediatric patients with hematuria are shown in Fig. 3.

### Other imaging techniques

X-rays of the abdomen are no longer routinely performed in children. In adult urology, CT has become the standard for stone imaging [28]. The appropriateness criteria for radiological imaging have recently been reviewed in detail. Ultrasound is the first and basic imaging modality for examining hematuria in children. Low-dose CT scans can also now be performed with a similar or lower amount of radiation than plain radiographs; however, in children, ultrasound is usually appropriate and sufficient to make a diagnosis [28]. In some exceptional cases, an appropriate CT scan may be required. In post-traumatic hematuria, contrast-enhanced CT is the best method of assessment, and delayed scans should be performed to detect abnormalities in the collecting system.

Urinary bladder hemangioma is a rare cause of gross hematuria in children. It often presents as isolated hematuria with eventual episodes of gross hematuria [30]. Multimodal imaging using ultrasound, CT, and magnetic resonance imaging (MRI) enables establishing the location and extent of the hemangioma while in unclear cases, cystoscopy may indicate a source of bleeding [31].

Bleeding is not uncommon in ADPKD due to mechanical damage to the cysts. In such cases, ultrasound follow-up is usually the appropriate procedure in children. In adult patients with ADPKD, total kidney volume can be assessed and monitored by CT or, preferably, MRI, which can help categorize patients, as well as monitor, evaluate, and assess the effectiveness of treatments such as tolvaptan aimed at slowing disease progression [27, 32].

### Cystoscopy

Cystoscopy is rarely used to assess hematuria in children. It can be indicated if a bladder mass is noted on ultrasound, or if posterior urethral valve or urethral abnormalities caused by trauma are suspected. In contrast to adults, bladder urothelial cell carcinoma (UCC) is an extremely rare cause of hematuria in children. However, patients after undergoing augmentation cystoplasty to treat neurogenic bladder (including ileocystoplasty, colocystoplasty, and gastrocystoplasty) are at increased risk for the subsequent development of cancer in the newly formed reservoir [33]. In these cases, knowledge of the risk and regular follow-up by ultrasound is needed. If ultrasound shows no abnormalities when UCC is suspected, cystoscopy should be considered for diagnosis [34].

### Performing a kidney biopsy

The decision to perform a kidney biopsy in children with asymptomatic hematuria is being re-evaluated in light of new clinical and genetic knowledge [23, 35].

There are several diseases where genetic testing has replaced kidney biopsy in establishing the diagnosis. Alport syndrome is the typical genetic disease presenting with hematuria in which genetic testing may replace biopsy if family history is suggestive [36].

It has been recommended that asymptomatic children with IMH should not routinely undergo kidney biopsy [5, 7]. In a study including 112 biopsies in asymptomatic children, minor glomerular lesions were found in those with IMH, while chronic glomerulonephritis (mostly IgAN) was the diagnosis when hematuria was accompanied by proteinuria. Many nephrologists perform kidney biopsies in patients with sub-nephrotic proteinuria (0.5 to 2 g/day), except when other circumstances may explain the latter. In a retrospective evaluation of kidney biopsies of 169 patients presenting with microscopic hematuria, the severity of glomerular lesions and the progression of kidney disease were closely related to urinary protein excretion [37]. Rapid deterioration of kidney function as well as clinical signs of autoimmune systemic disease would also emphasize the need of a kidney biopsy [38].

Depending on the assumed clinical diagnosis, certain targeted tests may be required prior to or in parallel with biopsy, including detailed analysis of possible auto-antibodies (antinuclear antibody (ANA), anti-neutrophil cytoplasmic antibody (ANCA), double stranded deoxyribonucleic acid (dsDNA), etc.), examination of the complement system, and exclusion of acute (e.g., Hanta virus) or chronic infections (hepatitis C virus).

The knowledge of kidney histology can significantly alter clinical management in patients with acute kidney injury (AKI) and help to determine the degree of active (potentially reversible) and chronic (irreversible) changes [38].

Periodic monitoring of hematuria and reassessment of the diagnosis should be carried out in the event of changes in clinical and laboratory data (increasing proteinuria, development of hypertension, decreasing kidney function).

## Some selected causes of glomerular hematuria

The incidence of the diagnosis of glomerular disease may depend on the “threshold” for performing a kidney biopsy [5]. In one study, IMH was associated with hypercalciuria (30–35%), hyperuricemia (5–20%), and glomerular disease such as IgAN and thin basement membrane nephropathy [6]. Kidney biopsy may help in assessing not only the type, but also the degree of disease activity [38]. Depending on local practices and ethnic differences, the prevalence or occurrence of the diagnosis from the biopsy may vary. The most common diagnoses on biopsy are IgA nephropathy (54%), Alport syndrome (25%), and acute postinfectious glomerulonephritis (APIGN) (13%) [7]. The common causes and characteristic clinical features of hematuria in childhood are shown in Table 2.

**Table 2:** Selected causes and characteristic clinical features of hematuria in children

Disease	Type of hematuria	Proteinuria	Other kidney manifestations	Other organ manifestations or symptoms
Immunoglobulin A nephropathy/IgA vasculitis nephritis (IgAVN)	In cases with kidney involvement: glomerular; less frequently, in the case of urological involvement: postglomerular	Variable, from normal to nephrotic range		In IgAVN palpable purpura, arthritis or arthralgia, bloody stool, neurological, genital and urological involvement
Alport syndrome	Persistent microscopic glomerular hematuria, eventually episodes with gross hematuria	From normal to nephrotic range		Family history, ocular manifestations, hearing loss, aneurysm, leiomyoma
Acute postinfectious glomerulonephritis	Microscopic or gross, glomerular hematuria	May reach nephrotic range	Arterial hypertension, acute kidney injury	In case of post-streptococcal nephritis, angina, eventually impetigo; 2 weeks of lag time
Nephrolithiasis	Gross hematuria in symptomatic cases, postglomerular hematuria		Kidney stones	Abdominal pain
Nutcracker syndrome	Gross and microscopic hematuria related to exercise, postglomerular hematuria	May be accompanied by proteinuria and be orthostatic in nature		May be associated with left flank pain, left-sided varicocele

### Immunoglobulin A nephropathy

IgA nephropathy (IgAN) and the histologically related IgA vasculitis nephritis (IgAVN, formerly Henoch-Schönlein purpura (HSP) nephritis) are collectively the most common causes of glomerulonephritis worldwide and a frequent cause of glomerulonephritis in children [39]. In Europe, IgAN is detected in 20% of children with glomerular diseases diagnosed by kidney biopsy [40]. IgAVN and IgAN both result from glomerular deposition of aberrantly glycosylated IgA1 but have different histological features and clinical courses. IgAN most often presents as slowly progressive mesangial lesions, while IgAVN presents as an acute episode characterized by inflammatory glomerular changes that may require immediate intervention to avoid chronic progression [41]. Their pathogenesis is complex and several different pathways are likely to be involved, interacting in a complex network [41] assuming the role of a common, in most cases unidentified, infectious trigger [40, 41].

IgAV is the most common vasculitis in children [39, 40]. Typical clinical symptoms include palpable purpura (without thrombocytopenia and coagulopathy), arthritis or arthralgia, and abdominal pain. Central nervous system involvement is a rare, severe, albeit reversible complication [42]. Rarely, urological complications may also occur and, in boys, scrotal pain may be a presenting symptom [43]. The ureter, bladder, prostate, testicles, and penis can also be involved, and may cause postglomerular hematuria. Nephritis (IgAVN) occurs in about 30% of patients with IgAV. The extent of kidney damage is the most significant prognostic element in determining morbidity and mortality [39, 40].

Hematuria may be microscopic, with episodes of gross hematuria occurring with or without a transient decrease in glomerular filtration rate during infective events. While hematuria is not a prognostic factor, even mild to moderate proteinuria may indicate severe glomerular morphological changes in IgAN on kidney biopsy. Kidney biopsy is usually only performed if the course shows a more severe disease with persistent proteinuria (> 500 mg/day) or increasing serum creatinine concentration [23, 44]. Previously regarded as a benign condition, a considerable percentage of patients will develop chronic kidney disease (CKD) and eventually progress to CKD stage 5 (CKD5) [45]. Progression may occur in about 20% of children who have been followed for at least 20 years [40]. Furthermore, CKD5 may occur in up to 15% of patients [45]. Of particular note is the prognostic significance of the Oxford classification as the relationship between the initial score results and the risk of progression to kidney failure remains unchanged across all age groups and decades after kidney biopsy [46, 47].

### Alport syndrome

Familial hematuria is a class of genetic disorders of the glomerular capillaries characterized clinically by persistent glomerular hematuria starting in childhood [48]. All patients with Alport syndrome (AS) and approximately 50% of those with the histological diagnosis of thin basement membrane disease (TBMN) have mutations in type IV collagen, the primary collagenous component of the glomerular basement membrane [48]. AS is caused by mutations in the *COL4A3*, *COL4A4*, and *COL4A5* genes, encoding the α3, α4, and α5 chains of type IV collagen, respectively [49]. X-linked recessive, autosomal recessive (AR), and, rarely, autosomal dominant (AD) modes of inheritance have been described. Nonmuscle myosin heavy chain IIA mutations have been identified as the cause of two rare forms of familial hematuria: the Epstein and Fechtner syndromes [50]. Assessing the family history of at-risk family members is important for timely identification of affected relatives and establishing the mode of transmission [6]. Male relatives with hematuria with kidney failure and hearing loss and female family members with hematuria must be identified. Clinical symptoms include kidney and ocular manifestations (lenticonus anterior and retinal changes), hearing loss, aneurysms of the thoracic and abdominal aorta, and leiomyomas [51]. Asymptomatic persistent microscopic hematuria is the first sign of kidney involvement in early childhood with normal serum creatinine and blood pressure. IMH hematuria may become macroscopic in the presence of intercurrent febrile illness, mimicking IgA nephropathy flare-ups. Proteinuria, hypertension, and progressive kidney failure develop over time [51].

### Acute postinfectious glomerulonephritis

Although the incidence of acute poststreptococcal glomerulonephritis (PSAGN) has decreased, it remains the most common cause of glomerulonephritis in children after IgA nephritis. PSAGN is an immunological complication of infection with group A β-hemolytic *Streptococcus*. The incidence of PSAGN is currently decreasing, presumably due to the successful treatment of streptococcal infections [52]. PSAGN manifests as microscopic or gross glomerular hematuria (red to brown urine), edema, proteinuria (rarely reaching nephrotic range), increased blood pressure, and AKI, with most commonly a self-limiting course [53]. Clinical presentation can vary from asymptomatic cases with microscopic hematuria to acute nephritic syndrome. Importantly, asymptomatic microscopic hematuria is the most common clinical finding. PSAGN is characterized by a temporary and significant reduction in the level of complement component C3. If, in addition to the persistence of symptoms of glomerulonephritis, the C3 level is permanently reduced, the possibility of C3 glomerulonephritis should also be considered in the differential diagnosis [52].

## Selected causes of postglomerular hematuria

The most common causes of postglomerular gross hematuria are urinary tract infection and hypercalciuria or nephrolithiasis [54]. Consequently, postglomerular causes account for the majority of hematuria assessed in the emergency department [28, 54]. The parallel detection of white blood cells (WBCs) likely suggests urinary tract infection [24]. It should be emphasized that using hematuria to predict the presence of urolithiasis has an accuracy of only 60% and the absence of hematuria does not rule out nephrolithiasis [54].

### Nephrolithiasis

During the process of formation of kidney stones, substances in the supersaturated urine precipitate and aggregate in the urinary tract or urinary bladder forming solid foreign bodies (kidney stones) [28]. The risk of stone formation is increased by elevated excretion of stone-forming compounds such as calcium, oxalate, phosphate, cysteine, and uric acid [54]. Typical ultrasound images are shown in Fig. 3.

The incidence of nephrolithiasis in children and adolescents is currently doubling every 10 years. The main causes for this increase are nutritional and environmental factors. Increased stone formation is seen in association with obesity and diabetes [28, 55]. In addition to excessive excretion of stone-forming substances, lower concentrations of inhibitors (magnesium, citrate) may also play a role in stone formation [56]. The role of the pediatric nephrologist in the diagnosis and management of nephrolithiasis has recently been extensively reviewed [28, 56]. While gross hematuria and abdominal pain are the presenting signs of symptomatic nephrolithiasis, asymptomatic patients may show up with incidentally discovered microscopic hematuria. Radiological diagnosis is based on ultrasound, while low-dose CT is only exceptionally used in children [30]. Metabolic factors such as calcium and citrate excretion, fluid intake as well as specific genetic diseases should be assessed in a systematic search for etiology [30, 55].

### Hypercalciuria

The most common cause of postglomerular IMH has been reported to be hypercalciuria (16–30%). It should be discussed as a separate entity since hypercalciuria presenting with signs of recurrent, isolated hematuria often precedes by years the development of overt nephrolithiasis [7, 57]. Hypercalciuria is defined as a urine calcium/creatinine ratio < 0.6 mmol/mmol (0.2 mg/mg) [58]. However, normal values may also vary depending on the age of the child and seemingly differ from region to region [59]. Hypercalciuria is a primary metabolic risk factor of kidney stones in children and may be associated with decreased bone density in addition to hematuria [57].

### Nutcracker syndrome

Compression of the left renal vein (LRV) by the superior mesenteric artery and aorta, causing renal vein congestion and resulting in hematuria, is aptly called nutcracker syndrome (NCS) [60, 61]. The proposed mechanism in explaining hematuria is that increased venous pressure into the LRV and left gonadal vein can lead to rupture of the septa between the venules and the collecting system in the kidney parenchyma. No glomerular damage has been reported in NCS [60]. It can also cause (orthostatic) proteinuria, left flank pain, but is regarded as a benign condition in most cases [29, 61]. An external sign suggesting the presence of NCS is a left varicocele in boys due to the formation of collaterals. NCS is relatively common in children with isolated hematuria and the inclusion of kidney Doppler ultrasound screening significantly improves the likelihood of making the diagnosis [29]. Management is determined by the severity of symptoms, which often resolve spontaneously over time [60]. NCS is suspected to correlate with a low body mass index (BMI) and can resolve with increasing BMI [62].

## New insights in the last 2 years: novel coronavirus disease 2019 (COVID-19)

SARS-CoV-2 (severe acute respiratory syndrome coronavirus 2) infection poses a new challenge for pediatric nephrology, as the disease itself can cause kidney damage or reveal a hidden kidney disease. Eventually, the same phenomena may also occur with the Pfizer-BioNTech COVID-19 vaccine (mRNA), which has been introduced for active immunization to prevent COVID-19 in individuals 5 years and older.

SARS-CoV-2 related AKI is the most common reported kidney damage, but other associations such as cases with macroscopic hematuria are also documented [63].

IgAN cases with crescentic glomerulonephritis with acute tubular injury have been described during COVID infection, with severe presentation and rapid progression to CKD stage 5 [63]. Furthermore, IgAN cases were also reported following administration of the Pfizer-BioNTech COVID-19 vaccine, but causal relationship still remains unclear [64]. In a case report, a teenage girl presented with gross hematuria and proteinuria within a few days after receiving the first and second dose of the Pfizer-BioNTech vaccine, although it changed to microscopic hematuria within 1 week. Previously, she had a 10-year history of microscopic hematuria [64]. Other cases have been reported where, after vaccination, macroscopic hematuria occurred in the remission phase of IgAN [65]. Pfizer-BioNTech COVID-19 vaccination may unmask previously undiagnosed glomerulonephritis in pediatric patients [66].

Thus, pediatric nephrologists should keep in mind that, during the COVID-19 pandemic and Pfizer-BioNTech COVID-19 vaccination period, the incidence of macroscopic or microscopic hematuria in patients with IgAN and/or chronic glomerulonephritis may increase [65]. Further investigations are needed to understand the underlying pathomechanism of such cases.

## Summary points

- The primary purpose of examining a child with blood in the urine is to determine the medical significance of the cause.
- It is essential to determine whether the blood is of glomerular or postglomerular origin.
- The combination of hematuria and proteinuria may be a sign of progressive kidney disease; thus, a detailed work-up is necessary.
- The incidence of the diagnosis of glomerular diseases in the case of hematuria may depend on the “threshold” for performing a kidney biopsy.
- The most common causes of glomerular hematuria are IgA nephropathy, Alport syndrome, and acute postinfectious glomerulonephritis. The most common causes of postglomerular gross hematuria are urinary tract infection, hypercalciuria, or nephrolithiasis.

## Multiple-choice questions (answers are provided following the reference list)

1. What is the most common cause of macroscopic postglomerular hematuria in childhood?
  - IgA nephropathy
  - Nutcracker syndrome, arteriovenous malformations
  - Nephrolithiasis, hypercalciuria
  - Bladder tumor
2. What is the prognosis of IgA nephropathy in childhood?
  - It usually leads to rapidly progressive glomerulonephritis
  - It is commonly a benign condition, but a considerable percentage of patients will develop chronic kidney disease
  - Is always a benign self-limiting condition
  - Its prognosis depends on the number of acquired infections in childhood
3. When would you indicate a kidney biopsy in childhood in the process of evaluating hematuria?
  - Hematuria cases with persisting acute kidney injury or chronic kidney injury with unknown origin
  - In every case of nephrotic syndrome
  - To verify Alport syndrome with a positive family background
  - In every case of persisting microscopic hematuria
4. What is the cornerstone of radiological imaging in suspected childhood nephrolithiasis?
  - Magnetic resonance imaging
  - Low-dose computed tomography
  - Ultrasound with the combination of cystoscopy
  - Ultrasound
5. Which of the following statements is true for hematuria in childhood?
  - Hematuria is a common finding in idiopathic nephrotic syndrome in childhood.
  - Macroscopic hematuria found in Henoch-Schönlein purpura always has an origin of bladder hemorrhage.
  - The combination of hematuria and proteinuria can be a sign of progressive kidney disease.
  - Hematuria during urinary tract infection is a hallmark of glomerular damage.

## Data Availability

Not applicable.
