# Publisher Correction: How to define and assess the clinically significant causes of hematuria in childhood

**DOI:** 10.1007/s00467-023-05978-y

**Published:** 2023-05-03

**Authors:** Orsolya Horváth, Attila J. Szabó, George S. Reusz

**Affiliations:** 1st Department of Pediatrics, Semmelweis University, 53-54 Bókay János Street, 1083 Budapest, Hungary

**Publisher Correction: Pediatric Nephrology**

https://doi.org/10.1007/s00467-022-05746-4

The PDF version of this originally published article was the uncorrected proof; it has now been replaced by the corrected version. The original article has been corrected.

